# Implementation of the Code of Marketing of Breast-Milk Substitutes in Vietnam: Marketing Practices by the Industry and Perceptions of Caregivers and Health Workers

**DOI:** 10.3390/nu13082884

**Published:** 2021-08-22

**Authors:** Tuan T. Nguyen, Ha T. T. Tran, Jennifer Cashin, Van D. C. Nguyen, Amy Weissman, Trang T. Nguyen, Bridget Kelly, Roger Mathisen

**Affiliations:** 1 Alive & Thrive Southeast Asia, FHI 360, Hanoi 11022, Vietnam; jcashin@fhi360.org (J.C.); aweissman@fhi360.org (A.W.); rmathisen@fhi360.org (R.M.); 2 Research and Training Center for Community Development, Hanoi 11616, Vietnam; ha.tran@rtccd.org.vn (H.T.T.T.); van.nguyen@rtccd.org.vn (V.D.C.N.); Trang.nguyen@rtccd.org.vn (T.T.N.); 3 Asia Pacific Regional Office, FHI 360, Bangkok 10330, Thailand; 4 School of Health and Society, University of Wollongong, Wollongong, NSW 2522, Australia; bkelly@uow.edu.au

**Keywords:** breastmilk substitutes, Code of Marketing of Breast-Milk Substitutes (the Code), infant, mixed-method study, Vietnam

## Abstract

Background: The promotion of breastmilk substitutes (BMS) is an important barrier to successful breastfeeding. Objective: To examine the enactment and implementation of the Code of Marketing of Breast-Milk Substitutes (the Code) in Vietnam with a focus on marketing practices by the baby food industry and perceptions of caregivers, health workers, and policy makers. Methods: From May to July 2020, we conducted a mixed-method, cross-sectional study including a survey of 268 pregnant women and 726 mothers of infants aged 0–11 months and in-depth interviews with a subset of interviewed women (*n* = 39), policy makers, media executives, and health workers (*n* = 31). Results: In the previous 30 days, two mothers (out of 726) participating in the quantitative survey reported that health workers had recommended BMS, at private hospitals in both cases. In-depth interviews with health workers showed that hospitals have internal procedures to prevent the promotion of BMS by health workers. However, companies employed representatives to promote products not covered under the Code (e.g., commercial milk formula for pregnant women) at antenatal care visits and by gaining contact information from women and using this information to promote BMS outside the hospital, often on social media. In the 30 days preceding the survey, one-fifth of pregnant women were exposed to promotions of commercial milk formula for pregnant women and 7.1% to promotions of BMS. Among mothers of infants, 7.3% and 10.7% of respondents with infants aged 0–5 and 6–11 months, respectively, were exposed to some form of BMS promotion in the past 30 days. Around the time of birth, parents commonly brought BMS to maternity facilities (52.5%) or purchased it nearby (35.4%). Conclusions: Although Vietnam has a strong regulatory environment for the protection, promotion, and support of breastfeeding, there are implementation, monitoring, and enforcement gaps. Stronger enforcement of national policies to regulate the presence of BMS industry representatives in health facilities—both public and private—and the promotion of BMS products on digital platforms are needed.

## 1. Introduction

The scale up of recommended breastfeeding practices to near universal levels could avert an estimated 700,000 maternal and child deaths and save the global economy over 341 billion USD each year [1]. Breastfeeding is nature’s perfect food system [2], and 95% of babies globally have ever received breastmilk: 97.6% in low-income, 95.6% in middle-income, and 78.8% in high-income countries [3]. Almost all mothers are biologically capable of breastfeeding their children; there is only one contraindication to breastmilk in children, the classic galactosemia—a rare congenital disorder of galactose metabolism—and there are only few conditions where mothers are recommended not to give their children breastmilk (typically due to the use of medication contraindicated in breastfeeding) [4]. However, recommended breastfeeding practices remain sub-optimal globally [5]. Aggressive marketing of breastmilk substitutes (BMS) is an important barrier to successful breastfeeding and poses a danger to child survival and health [6,7]. Previous studies have shown that inappropriate marketing of BMS affects breastfeeding behavior as well as the provision of breastfeeding support by health workers [8,9,10].

Initially adopted in 1981, the International Code of Marketing of Breast-Milk Substitutes [11] and subsequent resolutions adopted by the World Health Assembly (the Code) guide the protection and promotion of breastfeeding through the regulation of the marketing of BMS, bottles, and teats. The Code prohibits all forms of promotion of BMS, including advertising, gifts to health workers, and the distribution of free samples [11]. It includes provisions related to the nutritional and health claims made on labels, prohibits images that idealize artificial feeding, and requires that labels carry messages about the superiority of breastfeeding over BMS and the risks of not breastfeeding [11]. The Code prohibits promotion in all settings, including health facilities and in the public domain, as well as by anyone, including health workers and BMS industry representatives [11]. The Code also prohibits marketing personnel from having direct or indirect contact of any kind with pregnant women or mothers of infants and young children [11].

As of 2020, 136 of 194 countries assessed had some form of legal measure related to the Code, and 44 had strengthened their regulations compared with an earlier report in 2018 [6,12]. However, only 79 countries have national legislation that includes provisions regulating the promotion of BMS in health facilities, 51 prohibiting the distribution of free or low-cost supplies within the health care system, and 19 prohibiting the sponsorship of scientific and health professional association meetings by BMS manufacturers [6]. According to this report, Vietnam is among 42 countries with national legislation classified as moderately aligned with the Code (with a score of 73 out of 100 points) [6]. Vietnam’s legislation on the Code is the third highest in the Association of Southeast Asian Nations (ASEAN) region, behind the Philippines, which is substantially aligned with the Code (score of 85) and Myanmar, which is moderately aligned with the Code (score of 74).

In Vietnam, protecting, promoting, and supporting breastfeeding have represented a critical component of national efforts to improve nutrition since the early 1990s [13,14]. Vietnam officially adopted the Code through Government decrees such as Decree No. 74/2000/NDCP in 2000 and Decree No. 21/2006/ND-CP in 2006 [13]. The National Assembly passed the Law on Advertisement in 2012 to prohibit the advertisement of BMS for children under 24 months old, complementary foods for children under 6 months old, and feeding bottles and pacifiers [15]. In 2014, the Prime Minister approved the guiding Government Decree 100 on the marketing and use of nutritional products for young children, feeding bottles, teats, and pacifiers [6,16]. Although the Law on Advertisement and Decree 100 (together referred to as the Code in Vietnam) encompass a majority of the Code’s provisions, regulations related to the distribution of informational and educational materials by the BMS industry and provisions for monitoring and enforcement are relatively weak [6]. After almost a decade of Code implementation in Vietnam, evidence on the potential impact of these policies and bottlenecks for successful implementation from the perspective of various stakeholders and beneficiaries is limited [17].

The global market worth of commercial milk formulas (CMFs) increased from USD 1.5 billion in 1978 to USD 55.6 billion in 2019, representing a 36-fold increase over a 40-year period [7,18]. The BMS industry employs aggressive marketing tactics, including market segmentation [18], cross-promotion [19,20], and nutritional positioning [21,22], to drive sales of CMFs across the life cycle and across generations [23]. Furthermore, companies use tactics to market CMFs for pregnant and lactating women (CMF-PW) that are prohibited for BMS, including promoting products in health facilities, providing free samples, sponsoring research, and engaging in national policy development [24]. The production, sale, and marketing of CMF-PW are increasing globally, with the highest momentum in Asia, where an estimated 40% of new CMF-PW products were launched between 2013 and 2019 [25].

To contribute to addressing these knowledge gaps, this study aimed to examine the implementation of the Code in Vietnam with a focus on perceptions and experiences of caregivers and health workers.

## 2. Materials and Methods

### 2.1. Study Design

This was a mixed-method, cross-sectional study, employing primary quantitative and qualitative data collection [26]. All data were collected in person from May to July 2020. More details about the study design, questionnaires, and in-depth interview guides can be found in our published research protocol [26].

### 2.2. Setting

Vietnam is a lower-middle-income country in Southeast Asia with a population of 97 million, around one-third of which (36%) live in urban areas. Life expectancy at birth is 75 years. Each year, there are almost 1.6 million live births. The rate of neonatal mortality, infant mortality, and under-5 mortality are 11, 16, and 21 per one thousand live births, respectively [27].

Researchers conducted the study in two provinces and one municipality to capture various levels of socio-economic development: Bac Ninh, a province that is transforming from a predominantly agricultural to a more industrialized province in Red River Delta Region (north); Binh Duong, a predominantly industrial province in the Southeastern Region (south); and Ho Chi Minh City (HCMC), the most populous city in Vietnam (south). The population of these provinces is: 1,380,000 for Bac Ninh, with about 28% living in urban areas; 2,460,000 for Binh Duong, with about 80% living in urban areas; and 9,040,000, for HCMC, with 80% living in urban areas [28]. In 2019, there were about 34,200 live births in Bac Ninh, 43,200 in Binh Duong, and 127,400 in HCMC [28].

### 2.3. Participants, Sampling, and Data Collection

For the quantitative survey, researchers interviewed 994 women of reproductive age, including 268 pregnant women and 726 mothers of infants aged 0–11 months (infants), using structured questionnaires. We employed a multiple-stage, cluster cross-sectional sampling design in each of the selected provinces [26]. Stage 1—Selection of districts: we listed all sub-districts under three categories: urban, rural, and industrial zone. Within each category, we randomly selected a district. Stage 2—Selection of clusters/Primary sampling unit: within each selected district, we listed all sub-districts and randomly selected 10 sub-districts (a total of 30 sub-districts/clusters in the three districts). Stage 3—Selection of participants: the research team selected pregnant women and mothers of infants aged 0–5 and 6–11 months in each sub-district/cluster using systematic random sampling [26]. The sample included both permanent and temporary residents (i.e., migrant workers). Quantitative data from pregnant women and mothers were collected electronically using tablets and uploaded daily to a secure cloud-based server. A data manager downloaded the data from the cloud-based server and conducted frequent data quality checks.

For qualitative data collection, we conducted 39 in-depth interviews, including 9 pregnant women and 30 mothers of infants aged 0–11 months. The approach for identifying women for in-depth interviews was described previously [26]. Researchers also conducted interviews with 31 stakeholders at the provincial level, including policy makers (*n* = 9), media executives (*n* = 3), and health workers (*n* = 19) who have knowledge, a role in implementing, or a responsibility related to the Code in Vietnam [26]. To ensure data quality, investigators listened to the first three in-depth interview recordings and provided feedback to the team.

To ensure data quality, 2 supervisors and 18 enumerators were trained by investigators from the Research and Training Centre for Community Development (RTCCD) and Alive & Thrive (A&T). Researchers were supervised by investigators at RTCCD and A&T, and data were collected following a research protocol [26].

On average, each quantitative or in-depth interview took 45 min to complete. At the end of each interview, we gave VND 100,000 (equal to USD 4.5) for an interview with a pregnant woman or mother and VND 200,000 (equal to USD 9.0) for an interview with a health worker to compensate for their travel expenses and time. The interviewees also had an option to receive a gift such as a raincoat or a parenting book.

### 2.4. Variables

For the quantitative survey, we adapted standardized questionnaires [26], including those developed by the Network for Global Monitoring and Support for Implementation of the International Code of Marketing of Breast-Milk Substitutes and Subsequent relevant World Health Assembly Resolutions (NetCode) [29] to examine exposure to promotion of CMF-PW and BMS among pregnant women and of BMS among mothers of infants. Four main categories of promotion were explored (1) the provision of advice or recommendations (virtual or in-person), (2) free samples, (3) coupons, and (4) gifts [26]. For women who were exposed to promotion, we asked about the source and location of promotion. For mothers of infants, we also asked about promotion during the perinatal period [26]. Although NetCode questionnaires use a recall period of 6 months, we asked about the exposure in the previous 30 days to reduce recall bias [26]. We added questions about CMF-PW to learn about marketing tactics relating to these types of products in Vietnam. Exposure to CMF promotion among pregnant women was defined when the women were exposed to either CMF-PW or BMS. We combined all types of CMFs for infants and toddlers to a category of BMS by stage and did not ask about CMF brands. Additionally, through the testing of the questionnaire, we found that women had difficulties identifying specialties or qualifications of the health workers. We combined them by the place the health workers worked (e.g., private or public health facility). These modifications helped us to have a manageable number of questions in the CMF promotion section in the 6-section questionnaires.

We collected socio-economic characteristics of participating pregnant women and mothers, including age (years), ethnicity (Kinh, the ethnic majority group in Vietnam, and other ethnicities), marital status (married or unmarried), education (never attended school, primary school, junior secondary school, secondary school, diploma, or postgraduate), and employment status (farmer, blue-collar, white-collar, and small trader or self-employed, and unemployed, housemaker, student, or other).

Qualitative interviews with key informants at the municipal or provincial level and from the health sector addressed the following topics: (1) Code enactment, monitoring, and enforcement; (2) perceptions of and experience with the Code; and (3) suggestions for improvement [26]. For women, we asked about (1) perceptions of and experience with the Code, (2) suggestions for improvement, and (3) sharing responsibilities related to childcare and domestic tasks [26].

### 2.5. Data Management and Analysis

Descriptive data analyses, with some stratification by the type of CMF among pregnant women and age of the infants among mothers of infants, were conducted using Stata, Version 13.0 (Stata Corp. LP, College Station, TX, USA). All qualitative interviews were recorded. Records were transcribed fully by an independent team. A deductive thematic approach was used to categorize findings into themes using NVivo 11. Illustrative quotes were drawn from thematic sheets.

## 3. Results

Overall, 268 pregnant women and 726 mothers of infants participated in the quantitative survey. Most women were of Kinh ethnicity (93.3% of pregnant women and 95.3% of mothers), married (99.2% of pregnant women and 98.9% of mothers), and living with husbands or partners (96.3% of pregnant women and 97.0% of mothers) (Table 1). Of the pregnant women surveyed, 58.2% had a secondary diploma or higher and 21.6% had a white-collar job; while among mothers of infants aged 0–11 months, 62.8% had a secondary diploma or higher and 23.1% had a white-collar job (Table 1).

### 3.1. Women Are Exposed to Promotion of Commercial Milk Formula through Multiple Channels and from Pregnancy

**Figure 1:**
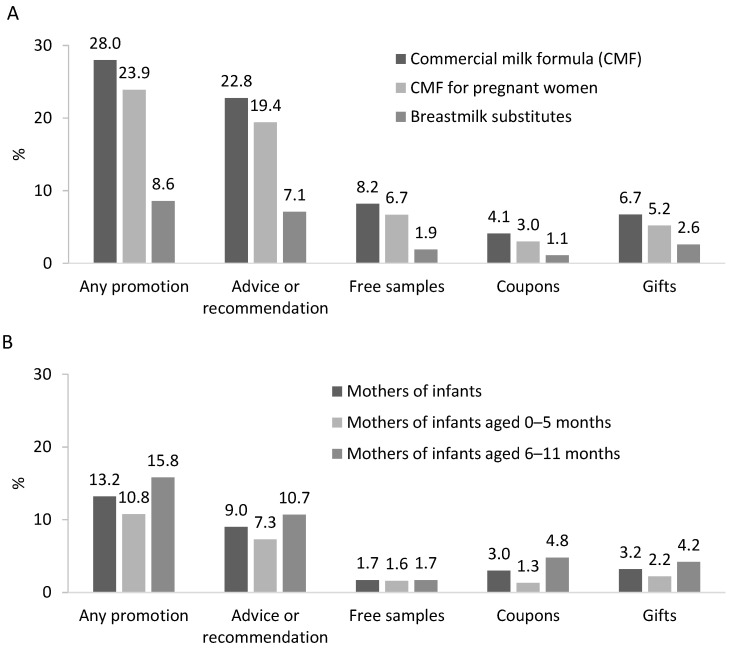
Exposure to promotion of commercial milk formulas (CMF-PW) and breastmilk substitutes (BMS) among pregnant women (**A**; *n* = 268) and BMS by mothers of infants aged 0–11 months (**B**; *n* = 726) in the previous 30 days.

In the 30 days preceding the survey, 28.0% of pregnant women were exposed to the promotion of CMFs (either CMF-PW or BMS), 23.9% to the promotion of CMF-PW, and 8.6% to the promotion of BMS (Figure 1). The most common promotion methods reported by pregnant women were the provision of advice or recommendations in person or virtually (22.8%) followed by the provision of a free sample (8.2%), other gifts (6.7%), and coupons (4.1%) (Figure 1). As reported by the 268 pregnant women, BMS industry representatives approached them during antenatal care visits at health facilities for personal information (16.0%), giving information advice on BMS (15.3%), or both (13.0%) (data not shown).

The prevalence of exposure to some forms of BMS promotion was 13.2% and was higher in mothers of infants aged 6–11 months (15.8%) than those with infants aged 0–5 months (10.8%) (Figure 1). Among mothers of infants, the most common promotion methods were advice or recommendations in person or virtually (9.0%), followed by the provision of gifts (3.2%), coupons (3.0%), and free samples (1.7%) (Figure 1).

**Figure 2:**
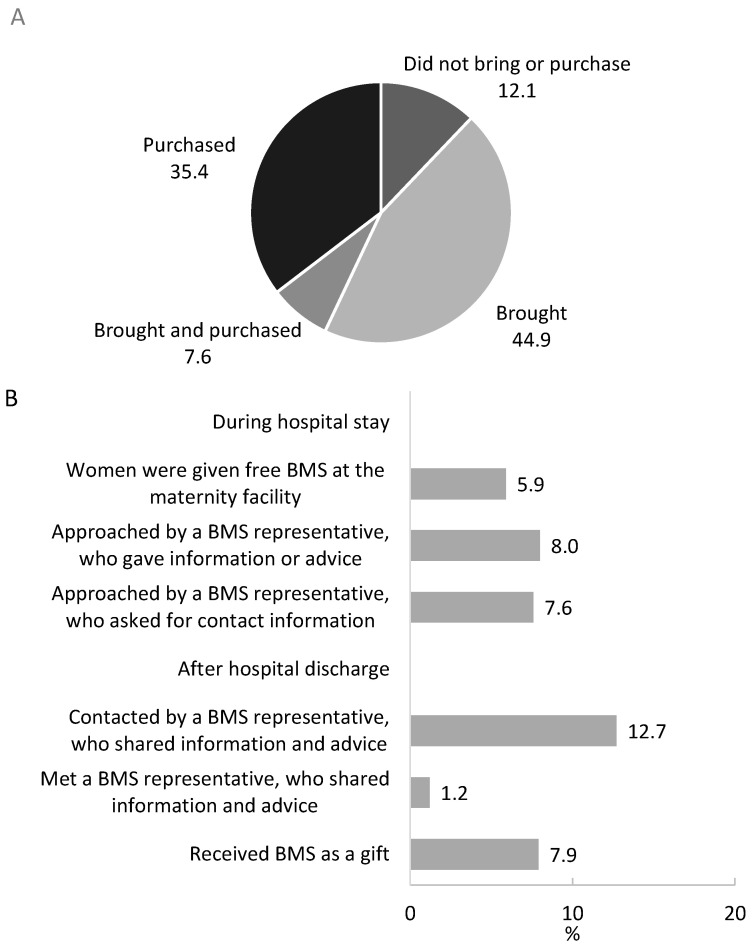
Bringing or purchasing of breastmilk substitutes (BMS) to the maternity facility at birth (**A**; *n* = 726) and exposure to BMS promotion during perinatal period (**B**; *n* = 726).

Women often bring or purchase BMS at maternity facilities at the time of birth. Among mothers of infants, only 12.1% of the women did not bring or purchase any BMS nearby their maternity facilities. A large number of women brought (44.9%), brought and purchased (7.6%), or purchased BMS (35.4%) within or nearby the facility (Figure 2). Mothers of infants also reported being contacted by BMS industry representatives during their hospital stay and after discharge (Figure 2).

In-depth interviews with mothers indicated that they have concerns about breastmilk supply in the first days after birth. As a result, the practice of bringing BMS with them to the hospital at the time of birth or buying it at shops near the hospital is common. This norm was reinforced by the availability of BMS within and nearby maternity facilities and health workers’ advice.

*When I went for my antenatal visit, peers instructed me about powdered milk for pregnant mothers. When approaching the due date, they recommended that I buy a can* [of formula milk] *to be ready in case I might not have breastmilk in the first day*.

Mother of 8-month-old child, HCMC.

*My family bought formula milk from the pharmacist’s shop at the gate of the hospital. The doctor advised us to buy formula milk for the child as she cried a lot and I seemed not to have breastmilk in the first 2 days*.

Mother in postpartum period in a public hospital, Binh Duong.

### 3.2. Health Workers Do Not Commonly Promote BMS at Health Facilities

In the previous 30 days, two mothers (out of 726) participating in the quantitative survey reported that health workers had recommended BMS, at private hospitals in both cases. In the previous 30 days, one pregnant woman (out of 268) received advice on using BMS from a health worker in a public hospital.

In-depth interviews indicate that hospital and health department leaders have a clear understanding of the Code and reportedly instruct staff in the obstetrics and pediatrics departments neither to sell BMS nor to have contact with BMS industry representatives or sales officers. Interviewed health workers confirmed that BMS industry had reduced their contact with staff in the obstetrics and pediatrics departments in recent years, as they were refused access by department heads and hospital leaders.

*The hospital’s policy prohibits formula milk companies from entering the hospital and promoting sales. If any [BMS] company wants to access the hospital facility, they must first contact the hospital’s Department of Administration and Planning. We review all aspects before granting such permission. In general, we do not permit the sale and advertisement of formula milk*.

Health manager, Bac Ninh.

However, in-depth interviews revealed that some health workers do not provide adequate breastfeeding support and encourage the use of BMS in health facilities.

*I bought formula at the hospital. The doctor asked me if my breastmilk had come out, then instructed me where to go and buy formula milk.*

Mother of 11-month-old child, Bac Ninh.

*I was hospitalized in the service department and the doctor came to counsel me about breastfeeding and how to choose formula milk for bottle feeding.*

Mother of 5-month-old child, Binh Duong.

During an in-depth interview with a mother of 2-month-old child, who happened to be the director of a company that sells BMS, she disclosed using current and former health workers to promote BMS and CMF-PW.

[Our approach entails] *introducing the formula to women via the advice of health workers, and these health workers are given a high commission. Families are more likely to trust and buy the brand* [if it is promoted by a health worker]. *Each health worker is assigned one brand. This way, I can identify the number of sales by each health worker*.

Mother of 2-month-old child, Bac Ninh.

In-depth interviews with health workers confirmed that some companies recruit former hospital staff to work as sales agents because they know where and when to best access mothers.

*I saw one former staff of the hospital; she visited the hospital with a laptop and talked with mothers in the wards. When I asked her what she did, she said she would not have a salary if she did not meet mothers and talk about formula milk, so I hesitated to ask her to leave. It is sensitive, you know*.

Health staff, Bac Ninh.

### 3.3. BMS Industry Representatives Promote Products within Health Facilities

**Figure 3:**
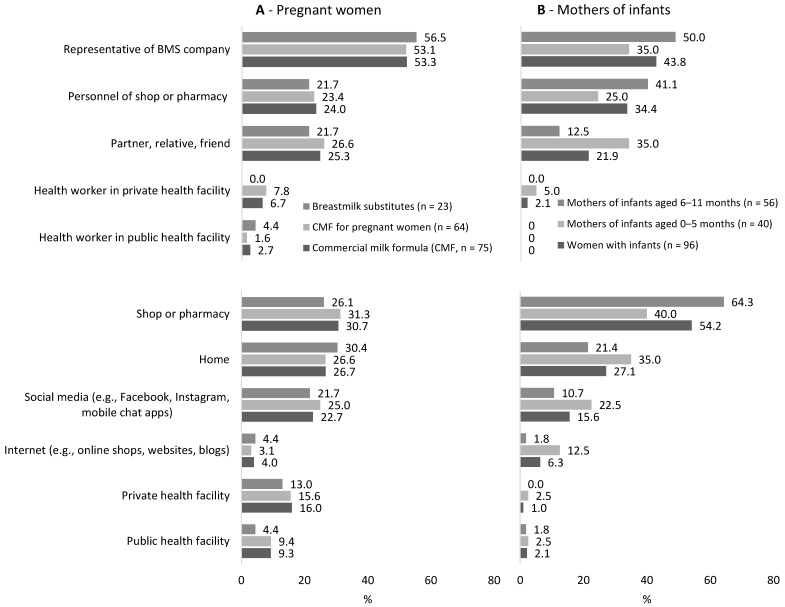
The sources and locations of the promotion of commercial milk formulas (CMF-PW) and breastmilk substitutes (BMS) to pregnant women (**A**) and BMS to mothers of infants (**B**), among those who were exposed to a promotion in the previous 30 days.

Among pregnant women who were exposed to a promotion of CMFs in the previous 30 days (*n* = 75), 16.0% and 9.3% reported the exposure in private and public health facilities, respectively (Figure 3). BMS industry representatives were the most common promoters of CMFs (53.3%), CMF-PW (53.1%), and BMS (56.3%) (Figure 3). Among those who were exposed to a promotion of BMS (*n* = 96), 43.8% of mothers of infants reported BMS industry representatives were the ones who promoted BMS (Figure 3).

Qualitative interviews with mothers and pregnant women reinforced the finding that BMS industry representatives approach pregnant mothers in hospitals, particularly while they are waiting for antenatal care visits. These representatives then obtain contact information (e.g., telephone number and account names on social media platforms such as Facebook and Zalo) and take photos or scan barcodes of women’s health record books in order to follow-up with women to promote BMS.

*When we were waiting for our antenatal care visit because they* [BMS industry representatives] *sat near the doctor, I thought they were health workers. There were two rooms. The room inside was the doctor’s room for antenatal examination. The room outside was for height and weight measurement, blood pressure measurement and health record filling. They sat in the room outside. They did not wear the hospital uniform but the milk company uniform.*

Mother of 8-month-old child, HCMC.

*While I was waiting for the antenatal care visit, a staff in uniform of a milk company approached me and asked for permission to sit down and talk to me. She asked my gestation week, number of children I had, whether I used any formula milk for pregnant mother, and my intentions related to feeding my child with breastmilk or formula milk. Then she asked for my permission to take a photo of my health record and scan the bar code on the record and asked for my phone number, and then made friends with me on Zalo* [a social media platform]. *Several days after, she called my phone while I was at home to inform me about a promotional campaign for milk for pregnant mothers and newborn formula and shared images of toys from the promotion, which I could get if I bought a large number of cans.*

Mother of 9-month-old child, HCMC.

Interviews indicated that mothers were also approached by shops and BMS industry representatives after birth, using their contact given during pregnancy. After discharge from the hospital, BMS industry representatives keep in frequent contact with mothers to inform them about promotional campaigns or to push further sales. A director of a milk trading company reported that his company used a digital system to estimate the average time it takes for an infant to consume all the formula milk purchased, which then drives the timing of advertisements and promotions.

*When I got my antenatal visit at the hospital … the Friso wholesale staff contacted me and advised about formula milk for child 0 to 1 year old. After my child was born, they shipped free samples of formula to my home*.

Mother of 5-month-old child, Binh Duong.

### 3.4. BMS Industry Promote Products on Mass and Social Media

Among pregnant women who were exposed to the promotion of CMFs in the previous 30 days (*n* = 75), the most common locations of exposure were at shops and pharmacies (30.7%), at home (26.7%), and on social media (22.7%) (Figure 3). The corresponding prevalence for the promotion of CMF-PW at the most common locations specifically were 31.3%, 26.6%, and 25.0% and for promotion of BMS specifically 26.1%, 30.4%, and 21.7%, respectively (Figure 3). Among mothers of infants who were exposed to the promotion of BMS (*n* = 96), the most common locations of exposure were at shops and pharmacies (54.2%), at home (27.1%), and on social media (15.6%) (Figure 3).

Findings from interviews with media executives suggest that the BMS industry have shifted their advertising budgets from television to social media. According to a key informant in Binh Duong, from 2013 to 2020, the total income from CMF-related advertisements on Binh Duong television reduced by about 10%–15% each year. However, advertisements for BMS and CMF-PW are still a reportedly significant source of income for local television companies.

*Milk companies will invest in a province based on the expenditure of the population. Binh Duong is on top of the revenue list for Vinamilk company, so they invested their advertisement budget to our province... In the last 10 years, all well-known formula companies have advertised their products here such as Abbott, Mead Johnson, Friesland Campina, Nestlé, Nam Yang... formula milk advertisement accounted for 60–70% of milk related revenue…According to our statistics, formula milk accounted for over 50% of our total revenue from advertisement in general (80 billion VND annually at least).*

Key informant, Binh Duong.

The BMS industry reportedly also uses social media to deliver tailored messages to market CMF-PW, leveraging women’s concerns for weight control. The information and advice were delivered in such a way that it was not perceived to be an advertisement, making them more likely to try the product.

*I scrolled through Facebook and saw an advisement about free samples of powdered milk for pregnant mothers. I registered and got free samples from Enfa and Friso during my first pregnancy. With this pregnancy, I logged into the community groups on Facebook and heard peers advising about Japan’s powdered milk for pregnant mothers which nurture the fetus but not make the mother fat. Therefore, I looked for and bought it.*

Mother of 4-month-old child, Bac Ninh.

Mothers also reported household and community influencing factors to feed their infants with BMS. For example, if they observed other infants in the neighborhood to be noticeably plump, they would ask their parents about which formula milk brand the baby was being fed. Mothers also reported pressure from family members to feed their infants with BMS.

*I think many mothers feed their children with formula milk due to pressure from the child’s grandparents and family members, not from the will of mothers. You will see a common situation in this province where grandparents told mothers “why is your child so tiny / small, why don’t you feed him/her with formula to grow better?”*

Mother of 6-month-old child, Bac Ninh.

## 4. Discussion

Vietnam has a strong regulatory environment for the protection, promotion, and support of breastfeeding [6,30]. Unlike in some other contexts [31,32,33], we found that the promotion of BMS is not primarily done by health workers at health facilities where they work. Health workers and hospital managers demonstrate a clear understanding of the Code and are not common promoters of BMS; thus, our study suggests that policies have been effective in this regard. Based on the Code in Vietnam [15,16], recommended breastfeeding practices (Criteria E1.3) have been integrated as one of 83 criteria under the National Hospital Standards and Accreditation for both public and private hospitals [34]. A hospital found to have Code violations will receive the lowest score (1 out of 5) of the Criterion E1.3 regardless of their performance in any other sub-criteria of this criterion [34]. Conversely, a maternity hospital with a score of at least 4 (good) on Criterion E1.3 is designated as a Baby Friendly Hospital [35] and eligible to pursue designation as a Center of Excellence for Breastfeeding [36] accredited by the Ministry of Health, which can help hospitals to attract more clients and increase their revenue. The inclusion of Code-related indicators within the national quality assurance system may have incentivized hospitals to comply with the Code and penalized the ones violating it. However, we also found that health workers in some small, private clinics and private hospitals promote BMS. Clinics unaffiliated with hospitals are not regulated under the National Hospital Standards [34] while private hospitals might have low motivation to meet the standards under criteria E1.3. Nonetheless, they are regulated by the Code in Vietnam, and our findings suggest that Vietnam needs to strengthen mechanisms for monitoring and enforcing the Code in these types of facilities.

Our study shows gaps in the implementation of the Code, which the BMS industry exploits to access pregnant women and mothers within health facilities and on social media. First, we found that BMS industry representatives promote products within health facilities. The BMS industry employs representatives who are not hospital staff, but who have medical backgrounds and connections within the hospital, to promote products. Findings from the qualitative interviews showed that while BMS industry representatives wear company uniforms, their presence in the health facility is accepted, allowing them to access women, begin to build relationships, and to collect contact information starting during early pregnancy. We found that women are willing to share their contact information if they perceive that the representative assisted them during the hospital or clinic visit or advised them on infant feeding. This tactic has been used by the BMS industry globally to understand and build long-term relationships with the potential customers [18,23]. The BMS industry leverages the power of association to increase its perceived respectability and connection to the medical field to promote products [23] by encouraging parents to associate BMS with medication and BMS industry representatives with health workers, most clearly evidenced by the employment of former government health workers as representatives or promoters [23,37].

The BMS industry uses CMF-PW as an entry point for accessing women and promoting BMS, justifying the presence of representatives in health facilities by promoting products that are not regulated by the Code [11,15,16,34]. Our findings show that the representatives promote BMS to pregnant women and obtain women’s contact information for further promotion and sales on social media. Promotion of CMF-PW is being used as an entry point for BMS promotion, as a part of a comprehensive strategy to build brand loyalty, cross-promote products, and drive sales across the life cycle—‘womb-to-tomb’ [18], and across generations [23]. Decree 100 (Article 12.2.c) states clearly that “Medical establishments cannot permit employees of establishments producing or trading in breastmilk substitutes to meet or contact nursing mothers and pregnant mothers in any forms” [16]. Thus, by Vietnam law, BMS industry representatives are prohibited from having contact with women in health facilities, regardless of what specific product they are promoting and whether it is covered within the scope of the Code [38]. These findings call for the need to better enforce the Code in Vietnam by fully restricting access to health facilities by the BMS industry and company representatives, including former health personnel, and regulating the promotion of CMF-PW.

Second, we found that more women were exposed to BMS promotion through digital media rather than traditional media channels, suggesting the Vietnam’s system for regulating Code violations on traditional media are largely effective. Digital marketing is a rapidly evolving field that applies increasingly innovative technologies to tailor ads and maximize interactions with consumers using multiple channels [18,39]. Globally, the food industry is shifting marketing budgets from traditional to digital media [39]. Such a shift in promotion strategies is likely to be effective in Vietnam because of the high level of internet infrastructure and literacy among Vietnamese people, especially in younger generations. In 2019, 64 million Vietnamese (66% of the 97 million population) were internet users and 62 million (64% of the total population) were active social media users, including YouTube (96%), Facebook (95%), and Zalo (74%) [40]. The average daily time spent using social media is 2.5 h or 37% of the average daily time spent using internet (6.5 h) [40]. In just a year, there was a marked increase in the number of internet users (add 6.2 million; or 4 percentage points) and active social media users (3 million, or 3 percentage points) [41].

Aggressive use of digital media channels to promote BMS to women has been reported earlier in Vietnam and other countries [18,23,37,38,42,43,44,45] and intensified during the COVID-19 pandemic [37,45]. Although the Code in Vietnam [15,16] regulates all channels of BMS promotion, including social media, the monitoring and enforcement of irrelevant ads and information on digital platforms is a challenge [23,38,46]. For example, in Vietnam, as of August 2020, although Google and Facebook hold 70% revenue of online advertisements, only 45% of revenue for ads on Google and 30% from Facebook are registered through media agencies registered in Vietnam, which are legally required to comply with local media regulations [46]. Furthermore, conflicting guidelines, inadequate monitoring and enforcement, as well as low penalties for detected violations hamper effective enforcement, even on advertisements registered through local media agencies [46].

Code violations on social media are difficult to regulate [18,23,37,46]. The BMS industry promotes products through company-sponsored influencers that spread advertisements in their networks and their followers’ networks through shares, likes, and hashtags [37]. Digital platforms collect and track information from users (e.g., their demographic information, socioeconomic status, and family information; search, view, like, and share data; and social networks) to optimize their marketing strategies such as information, pop-up ads, customer services, and peer support [23,37,39,44]. Furthermore, when a BMS industry representative collects personal information from pregnant women and mothers, he/she or the company can privately interact with the women as a friend, which is not feasible to regulate [15,16,18,23,39]. Social media platforms and the BMS industry should be held accountable for these violations [8,23,37].

There is also a need to raise public awareness on the Code, mobilize community monitoring, and use new technologies for monitoring, identifying, and censoring violations and enforcing the Code [37,46]. For instance, countries should use the artificial intelligence (AI) that Facebook, YouTube, and Twitter are already using to censor content [47] for Code monitoring and enforcement. In recognition of the potential for digital advertising and marketing to violate or abuse a child’s rights, the Committee on the Rights of the Child calls on governments to prohibit the use of children’s data and information to target potentially harmful commercial products (such as BMS) [18,23,48]. We argue that the use of a child’s birthdate and personal health information, scanned from a bar code on a health record or taken from a parent’s social media profile, constitutes targeting and is therefore a violation of child rights.

Third, routine exposure to BMS promotion in various channels can undermine breastfeeding and influence social norms in favor of artificial feeding [7,23,49,50,51]. Similar to other studies in the region and globally [23,32,50,52,53,54], we found that the BMS industry aggressively markets BMS products to women during pregnancy, at the time of birth, and during the postpartum period both in-person and on social media. The fact that almost 90% of the women brought BMS to the maternity facility at the time of childbirth or purchased it at or near by the facility suggests insufficient self-efficacy and unsupportive social norms related to the sufficiency of breastmilk in the first days of an infant’s life. The findings suggest the need for improving breastfeeding counseling for mothers and other family members during antenatal care, at the time of birth, and in the early days after birth, and for supporting breastfeeding through the provision of immediate and prolonged skin-to-skin contact, non-separation of the mother-baby dyad, and by reducing unnecessary cesarean sections [34,35,55,56,57].

Selected studies suggest that increasing father’s involvement and ensuring that mothers have a birth companion of choice are also effective at improving breastfeeding rates [58,59]. Building the supportive environment within and around health facilities [30] and making breastfeeding normative [51,60,61] are critical. Previous studies found that mass media campaigns, one-on-one and group counseling, and peer-support initiatives increased breastfeeding practices and improved social norms in Vietnam [62,63,64]. For at-risk newborns, including premature, low birthweight, and sick infants who cannot access their own mother’s milk, human milk bank services can help to ensure access to life-saving pasteurized donor human milk in the first few days of life [4,65,66].

Our study has several strengths, including the use of a mixed-method approach and a standardized questionnaire adapted from validated sources, including from NetCode [29]; although to minimize recall bias, we shortened the recall time for exposure to BMS promotion from 6 months to 30 days. Enumerators were thoroughly trained and used hand-held devices, which helped to reduce errors during data entry and collection, facilitated data checking at the central level, and sped up analysis. Few studies have collected and analyzed both qualitative and quantitative data related to the Code from women in diverse provinces in Vietnam. Including in-depth interviews with health workers allowed us to gain insights about the promotion of BMS within the health system.

We also acknowledge several limitations to our study, including the cross-sectional design with descriptive data analysis, which provides a snapshot of the situation in purposively selected provinces of Vietnam. The study design limits the generalizability of the findings on the inappropriate marketing practices in Vietnam or elsewhere. However, given that the BMS market leaders in Vietnam are multi-national companies, it is likely that the marketing tactics used in one context are also applied in other contexts, countries, and regions. In-person data collection occurred during the COVID-19 pandemic, which made some women hesitant to participate in the interview (non-response rate of 14.6% in the initial enquiry for the meeting). During data collection, restrictions on group activities, events, and access to health facilities could have led to an underestimation of the interpersonal, face-to-face promotion of BMS in the findings and an overestimation of the promotion on digital platforms.

## 5. Conclusions

Although Vietnam has a strong regulatory environment for the protection, promotion, and support of breastfeeding, there are gaps in the implementation, monitoring, and enforcement, which BMS industry representatives are exploiting to promote their products within health facilities and to the public. Stronger enforcement of national policies to regulate the presence of BMS industry representatives in health facilities—both public and private—and the promotion of BMS products on digital platforms are needed.

## Figures and Tables

**Table 1:** Socio-economic characteristics of women ^1^.

	Pregnant Women (*n* = 268)	Mothers of Infants Aged 0–11 Months		
		Total (*n* = 726)	0–5 Months (*n* = 372)	6–11 Months (*n* = 354)
Ethnicity, majority Kinh	93.3	95.3	95.4	95.2
Age (Mean ± SD)	29.3 ± 5.9	29.7 ± 5.5	29.5 ± 5.5	30.0 ± 5.5
Marital status, Married	99.2	98.9	98.7	99.2
Living with husbands, partners	96.3	97.0	96.2	97.7
Highest level of education				
Never attended school	1.9	1.9	1.6	2.3
Primary school	15.7	13.4	13.4	13.3
Junior secondary school	24.2	21.9	21.2	22.6
Secondary school	23.1	26.6	26.9	26.3
Diploma	16.8	17.9	19.1	16.7
Bachelors or higher	18.3	18.3	17.8	18.8
Main occupations				
Farmer	1.1	1.1	0.8	1.4
Blue-collar	18.3	30.2	32.0	28.3
White-collar	21.6	23.1	23.1	23.2
Small trader, self-employed, small self-owned business, services	34.0	27.3	24.5	30.2
Unemployed, homemaker, student	25.0	18.3	19.6	16.9

^1^ Data were presented as % or mean ± SD when specified.

## Data Availability

Requests for data may be directed to the corresponding author and are subject to institutional data use agreements.
